# Let the sun shine in: effects of ultraviolet radiation on invasive pneumococcal disease risk in Philadelphia, Pennsylvania

**DOI:** 10.1186/1471-2334-9-196

**Published:** 2009-12-04

**Authors:** Alexander NJ White, Victoria Ng, C Victor Spain, Caroline C Johnson, Laura M Kinlin, David N Fisman

**Affiliations:** 1 Child Health Evaluative Sciences, Research Institute of the Hospital for Sick Children, 123 Edward Street, Toronto M4V 1X6, Canada; 2 Faculty of Medicine, University of Toronto, 1 King's College Circle, Toronto M5S 1A8, Canada; 3 Dalla Lana School of Public Health, University of Toronto, Toronto M4T 3M7, Canada; 4 Division of Disease Control, Philadelphia Department of Public Health, 500 South Broad Street, Philadelphia 19146, USA; 5 National Centre for Epidemiology and Population Health, The Australian National University, Canberra 0200, Australia

## Abstract

**Background:**

*Streptococcus pneumoniae *is a common cause of community acquired pneumonia and bacteremia. Excess wintertime mortality related to pneumonia has been noted for over a century, but the seasonality of invasive pneumococcal disease (IPD) has been described relatively recently and is poorly understood. Improved understanding of environmental influence on disease seasonality has taken on new urgency due to global climate change.

**Methods:**

We evaluated 602 cases of IPD reported in Philadelphia County, Pennsylvania, from 2002 to 2007. Poisson regression models incorporating seasonal smoothers were used to identify associations between weekly weather patterns and case counts. Associations between acute (day-to-day) environmental fluctuations and IPD occurrence were evaluated using a case-crossover approach. Effect modification across age and sex strata was explored, and meta-regression models were created using stratum-specific estimates for effect.

**Results:**

IPD incidence was greatest in the wintertime, and spectral decomposition revealed a peak at 51.0 weeks, consistent with annual periodicity. After adjustment for seasonality, yearly increases in reporting, and temperature, weekly incidence was found to be associated with clear-sky UV index (IRR per unit increase in index: 0.70 [95% CI 0.54-0.91]). The effect of UV index was highest among young strata and decreased with age. At shorter time scales, only an association with increases in ambient sulphur oxides was linked to disease risk (OR for highest tertile of exposure 0.75, 95% CI 0.60 to 0.93).

**Conclusion:**

We confirmed the wintertime predominance of IPD in a major urban center. The major predictor of IPD in Philadelphia is extended periods of low UV radiation, which may explain observed wintertime seasonality. The mechanism of action of diminished light exposure on disease occurrence may be due to direct effects on pathogen survival or host immune function via altered 1,25-(OH)_2_-vitamin-D metabolism. These findings may suggest less diminution in future IPD risk with climate change than would be expected if wintertime seasonality was driven by temperature.

## Background

Many infectious diseases of public health importance exhibit predictable periodicity, with major increases in incidence during a specific season of the year [1]. Empirical evidence of such "seasonality" has been noted by physicians for centuries, and has been prominent enough to become a part of our vernacular (e.g., "cold and flu season") [1,2]. Despite this wealth of experiential evidence, the mechanisms underlying seasonality are poorly understood, especially diseases characterized by person-to-person transmission [1].

Invasive bacterial disease due to *Streptococcus pneumoniae *and other respiratory pathogens exhibits striking seasonality in its occurrence [3-7]. Pneumococcal infections are a common cause of severe, community-acquired illnesses, including community-acquired pneumonia requiring hospitalization, bacteremia, and meningitis [8]. While the introduction of antibiotics dramatically reduced the case fatality rate (CFR) for pneumococcal disease, the current CFR for bacteremic pneumococcal disease is still estimated at 5-10% in the United States, and may be twice as high among the elderly and in cases of meningitis [9,10]. The emergence of antimicrobial resistance to beta-lactam agents, macrolides, and other antibiotic classes is an important clinical concern [11]. Although the introduction of conjugate pneumococcal vaccines has been associated with a reduction in disease incidence [12], the recent increase in invasive infection by non-vaccine serotypes [13], which may be highly resistant to commonly-used antimicrobials [14], suggests that this microorganism will persist in challenging both the medical and public health communities.

The incidence of IPD peaks in the winter months, with annual periodicity [4,5], but the forces that drive this characteristic seasonality are unknown. Wintertime seasonality of communicable respiratory diseases are often assumed to be driven by seasonal changes in environmental conditions (e.g., diminished ultraviolet radiation (UV) exposure, decreased temperature) but such associations may be confounded by other seasonally varying factors, including population behaviours (e.g., clustering indoors), co-occurrence of other infections (e.g., influenza) [4], and frequency of laboratory testing [15].

A more thorough understanding of the effect of environmental factors on seasonal IPD incidence could offer significant insight into pathogenesis, improve disease forecasting, and help determine the likely direction of pneumococcal disease incidence in the face of global climate change [16]. Our objective was to investigate how environmental factors influence IPD occurrence in Philadelphia County. We used both traditional analytic methods (i.e., Poisson regression with seasonal smoothers) and a novel case-crossover method to examine the effects of acute weather fluctuations on IPD occurrence. Both methods reduce confounding by environmental, behavioural, and infectious influences that might otherwise distort the observed magnitude of environmental effects on disease risk.

## Methods

Philadelphia County encompasses an area of 369 km^2 ^in south-eastern Pennsylvania, and is coterminous with the City of Philadelphia (population 1,517,550 in the year 2000 [17]). The population receives public-health services from the Philadelphia Department of Public Health (PDPH). IPD has been a notifiable condition in the Commonwealth of Pennsylvania since 2002; Pennsylvania uses the uniform case definition endorsed by the National Notifiable Diseases Surveillance System [18]. A case is considered "confirmed" when a consistent clinical syndrome occurs in association with the isolation of *S. pneumoniae *from a normally sterile site (e.g. blood, cerebrospinal or pleural fluid). Data on IPD case occurrence in Philadelphia was obtained from PDPH records, and included date of onset, age, sex, race and ethnicity of the patient, isolation site, and fatal outcome (if known).

### Environmental Exposure Data

Meteorological data including temperature, relative humidity, wind speed, atmospheric pressure, and precipitation for the period from 2002 to 2006 was obtained from the weather station at Philadelphia International Airport, located eight kilometres southwest of Philadelphia's city center [19]. Information pertaining to air quality in Philadelphia County during the years of interest-including concentrations of lead, ozone, sulphur oxides and particulate matter-was obtained from the Environmental Protection Agency [20]. Because daily readings were taken at various locations throughout the region, the arithmetic means of the air quality values were used as exposure variables. UV index forecast estimates for Philadelphia during the same period were retrieved from the National Weather Service Climate Prediction Center [21]. Clear-sky UV indices represent an integral of measured UV radiation levels weighted by the ability of the different UV wavelengths to cause skin erythema. The issued UV index is a similar measure, which accounts for the effect of clouds on radiation transmission; because of inconsistencies in cloud measurement during the study period, we used the clear-sky UV index as our exposure variable.

### Statistical Methods

Rates of invasive pneumococcal disease were calculated using demographic data for Philadelphia County from the year 2000 US Census, as well as 2006 population estimates from the Bureau of the Census, with linear interpolation and extrapolation used to generate estimates for population by age and sex in other years [17]. We evaluated the seasonality of disease occurrence through construction of periodograms and autocorrelograms [15,22] for weekly case counts. As yearly periodicity was observed, we estimated seasonal and year-on-year trends in IPD occurrence using Poisson regression models that incorporated sine and cosine oscillators, with 52 week (annual) frequencies (i.e., incorporated fast Fourier transforms) [7,22].

Using these parameters, the expression for the expected number of case counts for a given week, *E *(*Y*) is given by:

where *cases *is an autoregressive model term reflecting the cumulative case count in the month prior to case occurrence, (i.e., *cases *= ).

The phase-shift of the composite waveform generated by combining sine and cosine components of the above equation can be approximated as the arctangent of *β*_2_/*β*_3_, and can be used to estimate the timing of peak disease incidence [22]. We also included model terms that controlled for longer term trends in incidence of invasive pneumococcal disease, which may have reflected the initiation of surveillance, the introduction of public funding for conjugate pneumococcal vaccination [12], changes in medical diagnostic practices, or other long-term changes in real or apparent pneumococcal epidemiology. As year-on-year trends in disease occurrence reflected a non-linear increase in disease risk, we used models that incorporated both linear and quadratic yearly terms.

The quadratic model term was statistically significant, but is difficult to interpret, thus we present our final Poisson model with separate linear yearly terms for the period from 2002 to 2003, and the period from 2004 to 2007 [23]. We evaluated the impact of environmental exposures on weekly IPD incidence by incorporating exposure variables into Poisson models both individually, and using a backwards elimination algorithm (with variables retained for *P *< 0.2 [24]).

To explore the possibility of effect modification by subject characteristics, we evaluated stratum-specific estimates of effect for age categories and genders. Heterogeneity of effects across strata was assessed using meta-analytic techniques, including both graphical inspection and calculation of meta-analytic *Q-*statistics [25]. We further explored the sources of between-stratum heterogeneity through construction of meta-regression models that estimate the contribution of group-level covariates to between-stratum variation in effects [25].

We used a case-crossover approach to evaluate acute (i.e., daily) associations between environmental exposures and IPD occurrence. This approach provides a means for evaluating the association between brief, transient exposures and rare outcomes. The design is characterized by "self matching", in that cases serve as their own controls. In the context of environmental epidemiology, a "case" is a day on which a case occurred, while a "control" is an appropriately selected day on which a case did not occur [6]. We used a time-stratified 2:1 matched case-crossover design in which hazard periods were defined as the reported date of IPD onset from Philadelphia County public health. Beginning on January 1, 2002 the person-time at risk was divided into three-week time strata. Control periods were chosen by matching the hazard period by day of the week within the stratum, and could both precede, both follow, or straddle the hazard period [26,27]. Random directionality of control selection was used in order to avoid biases that can occur with unidirectional or uniform bidirectional control selection [26]. The 1-3 day incubation period of *S. pneumoniae *was used to estimate the lag days between acute environmental occurrence and case onset, or plausible effect period [28]. We also evaluated effects during the period immediately preceding incubation (i.e., 4-6 day lags) to evaluate the possibility that environmental conditions might affect risk via enhanced transmission of *S. pneumoniae*. We evaluated the effects of both raw environmental exposures, and quantile ranks within time strata through construction of conditional logistic regression models [24]. Analyses were performed using SAS version 8.01 (SAS Institute, Cary, NC) and Stata version 9.1 (Stata Corporation, College Station, TX).

## Results

### Descriptive Epidemiology

Between January 1, 2002 and April 30, 2007 there were 602 reported cases of IPD in Philadelphia County, for a crude annual incidence of 6.84 cases per 100,000 [95% CI 6.32 to 7.42]. Incidence of IPD was highest at the age extremes, with a slight male:female predominance (incidence rate ratio (IRR) 1.16, 95% CI 0.99 to 1.36); the case fatality rate was approximately 10% (Table 1).

**Table 1:** Epidemiology of Invasive Pneumococcal Disease in Philadelphia County, 2002-2007.

Age	Cases	Incidence per 100,000 Person-Years of Observation
0 to 4	55	9.67
5 to 14	12	0.92
15 to 24	13	0.98
25 to 39	74	3.80
40 to 59	254	12.21
60 to 79	136	11.08
80 and over	58	16.74

**Gender**		

Male	302	7.39
Female	300	6.37

Died	53 (9.46%)	-

Total	602	6.84

**Figure 1:**
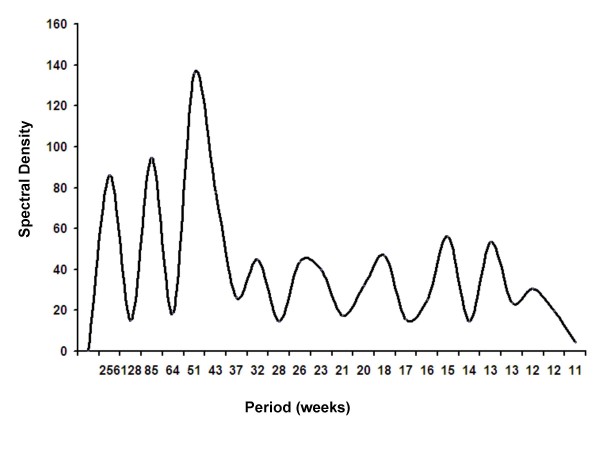
**Periodogram Constructed from Spectral Decomposition of Weekly Pneumococcal Case Counts**. Spectral density is represented on the *y*-axis, and can be conceptualized as a measure of goodness-of-fit for oscillatory regression models at different frequencies. The large peak at a frequency of 51 weeks suggests that invasive pneumococcal disease is a process that oscillates with annual periodicity (and is, in other words, compatible with wintertime seasonality). The two peaks at lower frequencies are lower harmonics illustrating bi- and tri-annual behaviour.

**Figure 2:**
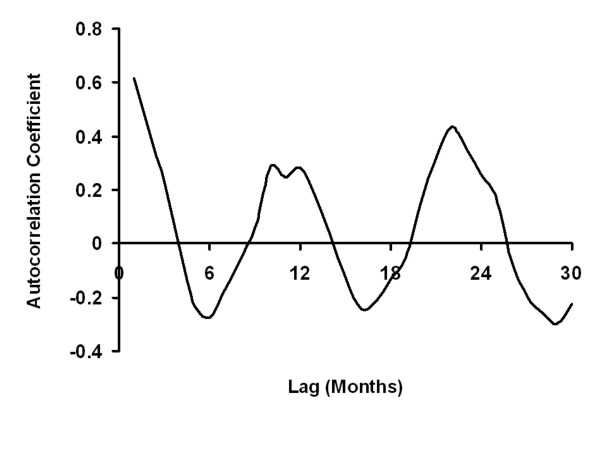
**Autocorrelogram for Weekly Invasive Pneumococcal Case Counts in Philadelphia, 2002 to 2007**. Positive autocorrelation is observed at intervals of 12 months, and negative autocorrelation is observed at intervals of 6 months, consistent with steady seasonal oscillation in disease risk.

**Figure 3:**
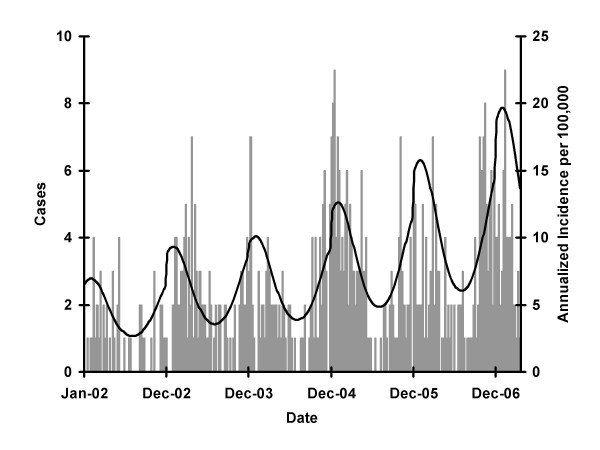
**Trends in Invasive Pneumococcal Disease in Philadelphia County**. Bars represent actual case counts, and the curve depicts expected incidence of disease occurrence based on a multivariable Poisson regression model including sine and cosine oscillators, an annual term, and a linear spline term with a knot at January 1, 2004. Reported cases are seasonal (wintertime predominant); incidence increases sharply in 2002 and 2003 with the introduction of mandatory reporting, and more slowly thereafter, as described in the text.

Both spectral decomposition and construction of autocorrelograms identified annual periodicity of infection, with peak incidence in mid-February (phase = 6 weeks) (Figures 1 and 2). Strong statistical evidence for seasonal oscillation was obtained from Poisson regression models (*P *for seasonal oscillation < 0.001). A significant annual increase in incidence was seen throughout the study period, though this was more marked prior to 2004 (IRR per year 1.34, 95% CI 1.08 to 1.66) than subsequently (IRR 1.22, per year 95% 1.16 to 1.34) (Figure 3). We found no clear trends in the incidence of IPD or case-fatality in individual age groups, and no significant heterogeneity was detected between age groups with respect to year-on-year trends in incidence or case-fatality (see Additional File 1 and Additional File 2).

### Weekly Environmental Effects

In univariable models the risk of IPD increased with several seasonally oscillating environmental exposures, including temperature, humidity, pressure, air pollution, and UV radiation, as shown in Table 2. Risk of IPD increased with average weekly barometric pressure, sulphur and nitrous oxides, and decreased with average weekly temperature, relative humidity, and UV index. However, after controlling for seasonal oscillation and longer term temporal trends, only cooling-degree days (i.e., average number of degrees above 18°C), maximum temperature, and clear-sky UV index were independently associated with case occurrencein a final multivariable model (Table 2). Risk increased with increasing maximal temperature, but decreased with cooling degree days, indicating a threshold of 18°C above which the qualitative effect of temperature changed.

**Table 2:** Weekly Weather Patterns and Incidence of Invasive Pneumococcal Disease in Philadelphia.

	Univariable Models			Multivariable Model^a, b^		
	
Environmental or Meteorological Exposure	IRR	(95% CI)	*P*	IRR	(95% CI)	*P*
Cooling Degree-Days (°C)^b^	0.92	(0.90 - 0.94)	< 0.001	0.97	(0.94 to 1.00)	0.054
Maximum Temperature (°C)	0.97	(0.96 - 0.97)	< 0.001	1.03	(1.003 to 1.06)	0.028
Minimum Temperature (°C)	0.96	(0.95 - 0.97)	< 0.001	...	...	...
Relative Humidity (%)	0.98	(0.97 - 0.99)	0.002	...	...	...
UV Index	0.89	(0.87 - 0.92)	< 0.001	0.70	(0.54 - 0.91)	0.007
Sulphur Oxides (ppm × 100)	1.73	(1.27 - 2.37)	0.002	...	...	...
Average Wind Speed (km/h)	1.01	(1.006 - 1.015)	< 0.001	...	...	...

**NOTE**: Incidence rate ratios (IRRs) reflect change in disease risk per unit change in the meteorological variable in question. CI, confidence interval; ppm, parts per million.

^a^The model was also adjusted for seasonal oscillation, cumulative cases during the prior month, calendar year and year-squared.

^b^Model deviance statistic chi-squared = 3156 on 9317 *d.f*., P = 1.000.

^c^Cooling degree days are defined as the number of degrees a daily mean temperature is above 24°C. For example, a day with an average temperature of 27°C would have 3 cooling degree-days.

**Figure 4:**
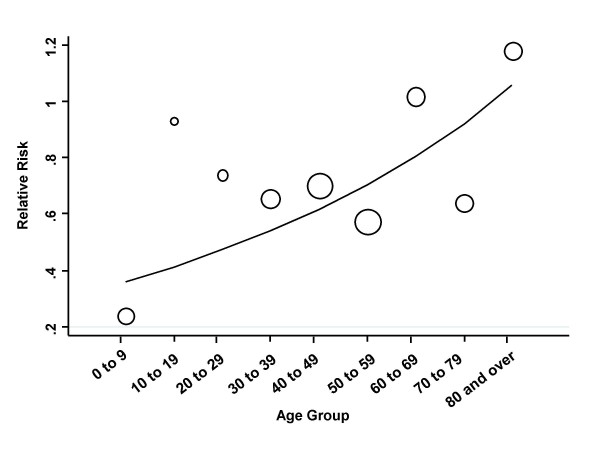
**Changing Effect of UV Radiation on Invasive Pneumococcal Disease Risk by Age Group**. Figure constructed by performing meta-regression using age-stratum specific estimates of the effect of a single unit increase clear-sky UV index on the incidence of invasive pneumococcal disease. A log-linear increase in the effect of UV index is observed with decreasing age.

There was significant heterogeneity in the effect of clear-sky UV index in individuals aged less than 15 years (IRR 0.26, 95% CI 0.11 to 0.61) as compared to older individuals (IRR 0.81, 95% CI 0.64 to 1.03), (*Q*-statistic 6.31 on 1 *d.f*., *P *= 0.012). No heterogeneity in UV effects was found when evaluated in individuals aged over 64 as compared to younger individuals (*Q*-statistic 0.48 on 1 *d.f*., *P *= 0.523), or when evaluated in males as compared to females (Q-statistic 1.68 on 1 *d.f*., *P *for heterogeneity = 0.194). Meta-regression models constructed using stratum-specific estimates of effect for age groups, by 10-year age increments, identified a log-linear trend in the effect of UV radiation on disease risk by age stratum, with strongest effects in youngest strata, and effects diminishing with increasing age (change in natural logarithm of IRR = 0.13 per decade, 95% CI 0.04 to 0.23, *P *= 0.007) (Figure 4).

### Acute Environmental Effects

Evaluating associations between environmental and meteorological exposures and IPD risk using a case-crossover approach, we identified an inverse association between ambient levels of sulphur oxides and disease risk during the likely incubation period (Table 3). No other significant associations between environmental exposures and risk were identified either during the incubation period, or in the period immediately prior to incubation. In particular, occurrence of IPD was not affected by daily changes in clear-sky UV index, temperature or cooling-degree days, in contrast to associations on longer time scales described above.

**Table 3:** Associations Between Acute Environmental Exposures and Invasive Pneumococcal Disease by Case-Crossover Analysis.

	Incubation Period (1-3 Days Prior to Case Occurrence)			Prior to Incubation Period (4-6 Days Prior to Case Occurrence)		
	
Exposure	OR	(95% CI)	*P*	OR	(95% CI)	*P*
*Meteorological*						
Cooling Degree-Days (°C)	0.878	0.624-1.236	0.456	0.926	0.676-1.270	0.635
Maximum Temperature (°C)	1.036	0.855-1.256	0.717	0.997	0.822-1.209	0.974
Minimum Temperature (°C)	0.994	0.820-1.204	0.948	1.039	0.858-1.258	0.694
Atmospheric Pressure (kPa)	1.026	0.848-1.241	0.794	1.013	0.834-1.230	0.895
Precipitation (mm)	0.954	0.783-1.162	0.6400	0.897	0.731-1.101	0.300
Relative Humidity (%)	1.038	0.844-1.275	0.725	0.989	0.804-1.217	0.916
UV Index	1.148	0.944-1.396	0.168	1.061	0.874-1.289	0.548
Average Wind Speed (km/h)	0.900	0.737-1.098	0.298	1.045	0.851-1.283	0.6739
*Air Quality*						
Sulphur Oxides (ppm)	0.747	0.603-0.925	0.007	1.425	0.952-1.425	0.138
Oxides of Nitrogen (ppm)	0.918	0.735-1.147	0.452	1.144	0.930-1.406	0.203
Carbon Monoxide (ppm)	0.925	0.751-1.139	0.461	0.996	0.809-1.227	0.971
PM10 (μg/m^3^)	0.215	0.732-3.999	0.215	1.199	0.523-2.750	0.668
Ozone (ppm)	1.018	0.830-1.249	0.861	0.904	0.730-1.118	0.351

NOTE: OR, odds ratio; ppm, parts per million; kPa, kilopascals; PM10, particulates 10 micrometers or less in diameter. Presented odds ratios are those associated with upper tertile of exposures during incubation period. No significant associations were detected in analyses using raw exposure data.

## Discussion

Notwithstanding the existence of vaccination and effective antibiotic therapy, invasive pneumococcal disease remains an important source of population morbidity and mortality. The seasonality of IPD is well recognized, but poorly understood. Epidemiological mechanisms invoked to explain this pattern have included co-occurrence of other infectious diseases [4], wintertime social gatherings [5], and seasonal oscillation in immune function [29]. However, concurrent seasonal changes in a variety of environmental, behavioural, and epidemiogical exposures make identification of causal associations particularly challenging [30]. We attempted to address this challenge by using analytic approaches that should control for seasonal confounders, known and unknown, at different time scales. At a weekly time scale we found increases in UV radiation to be most strongly associated with decreased numbers of invasive pneumococcal disease cases, though average temperatures also appeared to influence disease risk. At short time scales, fluctuations in ambient air quality, as manifested by differences in concentrations of sulphur oxides, were associated with changes in risk. The direction of this association was at variance with existing models relating air pollution to pneumonia occurrence [3,31,32].

The casual (as opposed to causal) association between low UV radiation in the winter and surges in respiratory disease has been noted previously [33], and has been proposed as an important driver of influenza seasonality, but has not to our knowledge been evaluated in a way that accounts for coincident seasonal changes in other seasonally oscillatory factors. The degree to which such seasonal oscillation can result in "just so" stories that lead to misattribution of causation to non-specific seasonal exposures is highlighted in the univariable analyses we conducted without including seasonal oscillators. In these models, a variety of environmental conditions, including weather variables and air quality indices, were strongly associated with IPD risk. However, after controlling for non-specific seasonality, only UV radiation (and, more weakly, temperature) were associated with disease risk; indeed, the apparent protective effect of UV radiation was actually strengthened after controlling for seasonal oscillation. The interpretation of such a model is that increases in UV radiation reduce IPD risk, even *after *accounting for the fact that IPD risk is maximal during low-UV periods of the year.

An important consideration is whether changes in UV radiation, operating at a weekly time scale, constitute a biologically plausible mechanism that explains seasonal oscillation in pneumococcal disease risk. Indeed, there are several mechanisms that may have substantial biological plausibility. Modulation of risk may occur through direct effects of UV radiation on host immune function: Dowell reviewed a variety of immunological changes associated with diminished UV radiation exposure in experimental settings, and noted that in granulocyte and monocyte function were reduced during periods of short light exposure [33]. UV radiation also influences the production of 1,25-(OH)_2_-vitamin D, which has important immunomodulatory functions [34]. Namely, enhanced maturation of macrophages, macrophage secretion of bactericidal substances such as lysozomal enzyme phosphatase and hydrogen peroxide [33,35], and secretion of antimicrobial peptides (including cathelicidins and defensins) by both immune cells, and respiratory tract epithelium [36]. Vitamin D deficiency is associated with a marked increase in the risk of pneumonia [37], and most human vitamin D is acquired via sun exposure. Thus, extended periods of low UV light could result in an increased susceptibility to *S. pneumoniae *infection resulting from a lack of vitamin D production, though the week-to-week fluctuations in risk described in this paper may be too rapid to represent a vitamin D effect.

An alternative mechanism of action of UV radiation in reducing IPD risk could be direct effects of radiation on pathogen survival. Many bacterial respiratory pathogens, including pneumococcus, are transmitted in respiratory secretions over short distances (i.e., via "large droplet transmission"), and thus encounter the physical environment directly during transmission events. The bactericidal effects of UV-B radiation have been well known for decades; such radiation inactivates bacteria by causing harmful genetic mutations through creation of pyrimidine dimers [38]. A model of UV effect via diminished transmissibility, rather than decreased host susceptibility, is supported by our finding that UV effect is strongest in the youngest individuals in the population (i.e., toddlers), where disease risk is likely to be driven by mobility, contact with peers, prolonged carriage and carelessness with respiratory secretions. We found very little protective effect against IPD in oldest individuals, whose risk may be more strongly linked to immune senescence than to high rates of contact with infectious contemporaries [39,40].

An unexpected finding in our case-crossover analysis was the identification of increased levels of ambient sulphur oxides with diminished risk of IPD. This may represent a chance association, due to the established association between sulphur dioxide and adverse respiratory outcomes [32], as well as the previously described correlation between ambient sulphur dioxide and pneumonia risk [3]. Nonetheless, this association may warrant further exploration; for example, ambient air pollutants might have adverse effects on respiratory tract pathogens as well as hosts.

Other findings in this study are also worthy of comment; in Philadelphia, the wintertime peak in pneumococcal incidence occurred about six weeks later than previously been described in U.S. adults [5]. In addition, a gradual increase in cases was observed during the 5-year study period. We suspect that this increase is less likely to represent a true surge in disease rates, which have actually been falling in the U.S. with the introduction of 7-valent conjugate pneumococcal vaccine [41]; rather, we suspect that this increase, which is more attenuated after 2003, represents the gradual increase in reporting that commonly follows implementation of new infectious disease reporting requirements [42].

Like any observational study, ours has several limitations. First among these is our heavy reliance on public health surveillance data which is known to suffer from under-reporting of notifiable infectious diseases [43]. Thus our data set may be incomplete, consisting of only a subset of the cases of IPD in Philadelphia during the study period. However, selection bias will only be introduced if the notification of infectious diseases to public health was correlated with meteorological patterns (e.g., cases which occur during days with higher UV index have a greater likelihood of being diagnosed and reported), which seems unlikely [44]. Second, we obtained weather data from a single site at Philadelphia International Airport, and air pollution and UV data were averaged over several locations throughout the county, which may not represent the true exposure status of individual cases. This is probable non-differential misclassification, and will bias the results towards the null hypothesis. The effects seen here are most likely conservative, weakening the strength of observed associations and suggesting our estimates tend towards the lower bound [44].

## Conclusion

In summary, we described the occurrence of IPD in a major U.S. urban center and found that incidence was associated with marked wintertime seasonality that may be partly explained by diminished exposure to UV-B radiation in winter months. Further study is needed, but this result is consistent with observed patterns of respiratory infectious disease, and would be consistent with several biologically plausible models of effect. As the nature of future changes in UV radiation related to climate change are more difficult to predict than general changes in temperatures or precipitation patterns, the implications of these findings for future pneumococcal disease epidemiology are unclear [45]. Nonetheless, we believe this observation goes some way towards explaining the notable seasonality of IPD, and could conceivably lead to novel disease control strategies through improved understanding of this common and virulent infectious disease.

## Competing interests

The authors declare that they have no competing interests.

## Authors' contributions

AW performed the majority of the analysis and interpretation of the data, and drafted the manuscript. CJ and CS acquired the case and exposure data, and each performed revisions of the manuscript. LK analysed and interpreted data, and was involved in drafting and revising the manuscript. VN adapted the analysis tools for the data, assisted in its interpretation and was involved in critically reviewing the manuscript. DF performed data analysis, interpretation, and was involved in both the drafting and critical review process of the manuscript. All authors have given approval for the final version to be published.

## Pre-publication history

The pre-publication history for this paper can be accessed here:

http://www.biomedcentral.com/1471-2334/9/196/prepub

## Supplementary Material

Additional file 1 Graph shows trends in invasive pneumococcal disease incidence by age group in Philadelphia from 2002 to 2007. No differences in trends are observed across age groups. Click here for file

Additional file 2 Graph shows trends in invasive pneumococcal disease case fatality rate (%) by age group in Philadelphia from 2002 to 2007. No differences in trends are observed across age groups. Click here for file
