# Supplementary figures and images for: Let the sun shine in: effects of ultraviolet radiation on invasive pneumococcal disease risk in Philadelphia, Pennsylvania
Source: BMC Infect Dis. 2009 Dec 4;9:196. doi: 10.1186/1471-2334-9-196 (PMC2797517; doi:10.1186/1471-2334-9-196)

## Slide 1
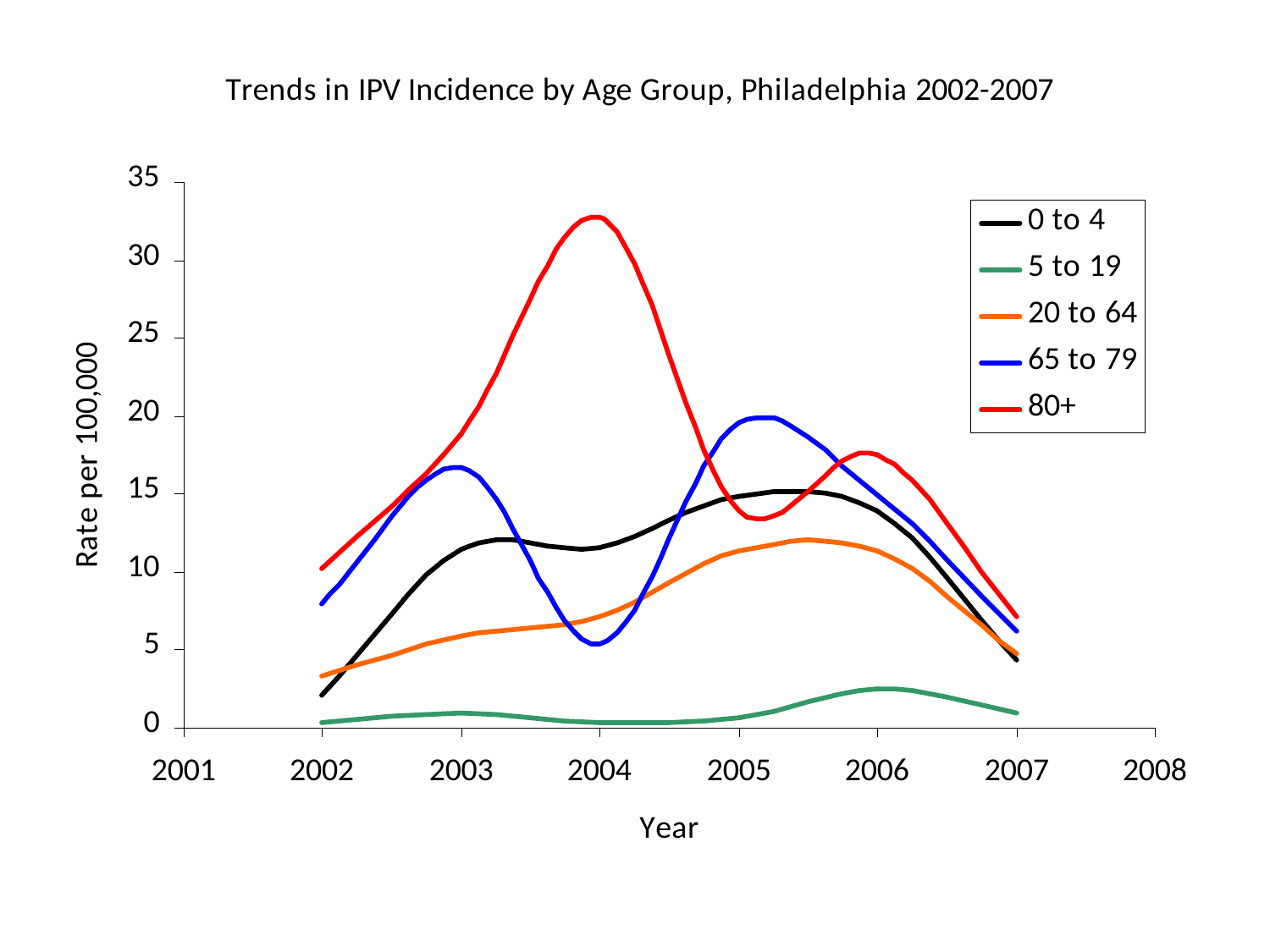

Supplement: Additional file 1 — Graph shows trends in invasive pneumococcal disease incidence by age group in Philadelphia from 2002 to 2007. No differences in trends are observed across age groups. [file 1471-2334-9-196-S1.PPT]

## Slide 1
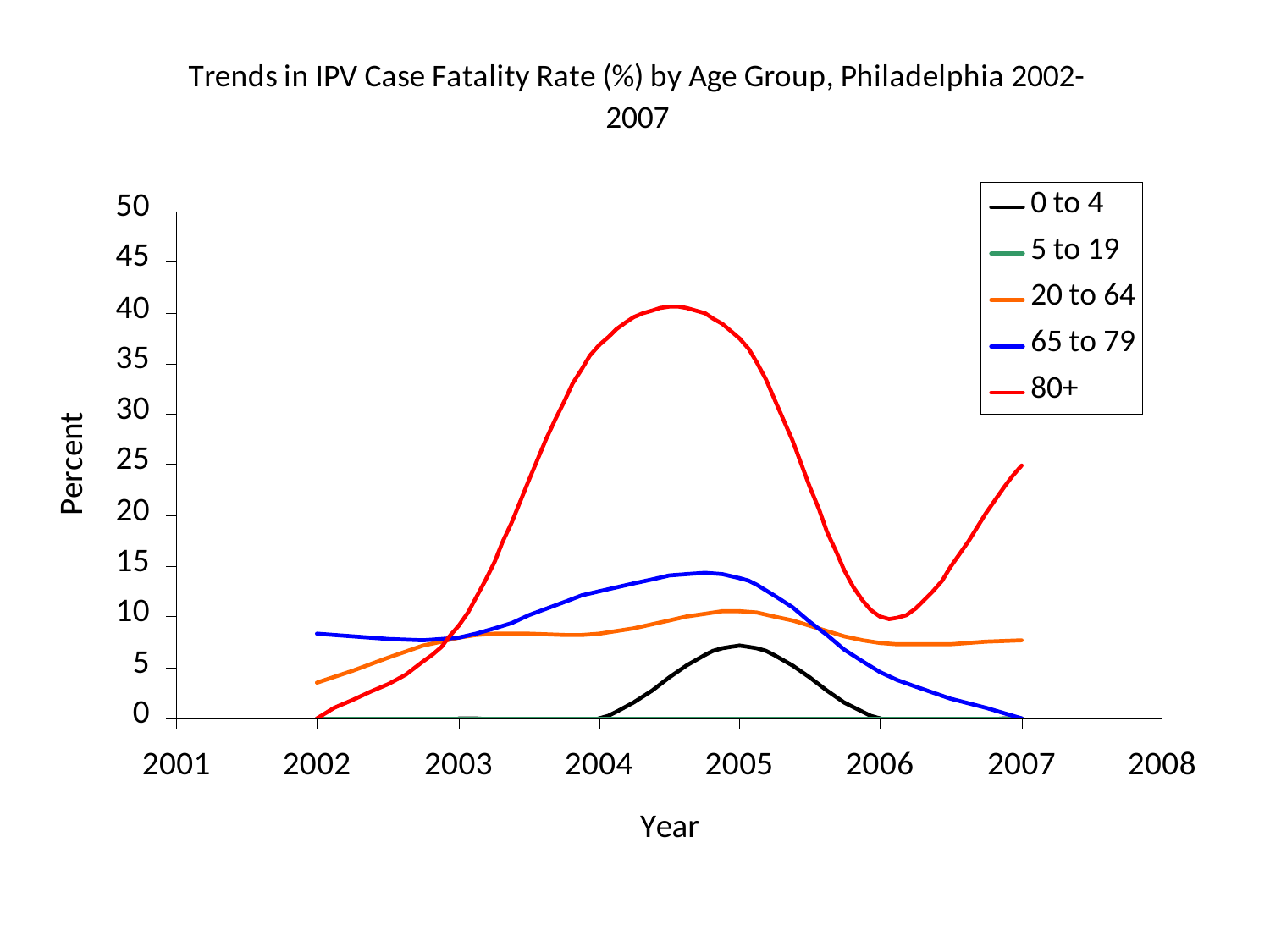

Supplement: Additional file 2 — Graph shows trends in invasive pneumococcal disease case fatality rate (%) by age group in Philadelphia from 2002 to 2007. No differences in trends are observed across age groups. [file 1471-2334-9-196-S2.PPT]
